# Maternal ABVD chemotherapy for Hodgkin lymphoma in a dichorionic diamniotic pregnancy: a case report

**DOI:** 10.1186/s12884-020-02928-6

**Published:** 2020-04-19

**Authors:** Camille Cotteret, Yen-Vi Pham, Ambroise Marcais, Marine Driessen, Salvatore Cisternino, Joël Schlatter

**Affiliations:** 1 Hôpital universitaire Necker - Enfants malades, Pharmacie, F-75015 Paris, France; 2 Hôpital universitaire Necker - Enfants malades, Hématologie adultes, F-75015 Paris, France; 3 Hôpital universitaire Necker - Enfants malades, gynécologie-obstétrique, F-75015 Paris, France; 4 Inserm UMR-S 1144, Team “Blood-brain barrier in brain pathophysiology and therapy”, Université Paris Descartes, F-75006 Paris, France

**Keywords:** Twin dizygotic pregnancy, Hodgkin lymphoma, Doxorubicin, Cardiotoxicity, Fetus

## Abstract

**Background:**

Hodgkin lymphoma (HL) is the most common hematological malignancy during pregnancy. The first-line treatment for HL in pregnancy is the standard ABVD regimen without any drug and/or dose adjustment. However, data on chemotherapy during twin pregnancies are sparse, and a better understanding of the mechanisms involved in exposure to and the toxic effects of anticancer drugs in the fetuses is needed.

**Case presentation:**

A 41-year-old dichorionic diamniotic pregnant patient was given ABVD treatment for HL at a gestational age of 28 weeks and 3 days. The patient received 2 cycles of chemotherapy with a 15-day therapeutic window including an actual 25 mg/m^2^ dose of doxorubicin per cycle. Unlike the female twin, the male twin presented four days after birth a left cardiac dysfunction. Doxorubicin cardiotoxicity in the male newborn was also supported by high blood levels of troponin. At one month of age, echocardiography findings were normal. We investigated literature data on physiological aspects of pregnancy that may influence doxorubicin pharmacokinetics, and pharmacodynamic and pharmacokinetic data on the use of doxorubicin in pregnancy. We detailed the role of the transporters in doxorubicin placenta distribution, and tried to understand why only one fetus was affected.

**Conclusions:**

Fetal safety depends at least on maternal doxorubicin pharmacokinetics.Because of drug interactions (i.e. drug metabolism and drug transport), care should always be taken to avoid maternal pharmacokinetic variability. The toxic effects were discrepant between the dizygotic twins, suggesting additional fetus-specific pharmacokinetic/pharmacodynamic factors in doxorubicin toxicity.

## Background

While the simultaneous occurrence of cancer and pregnancy is a rare medical event, hematological malignancies such as Hodgkin lymphoma (HL) occur in about 1/6000 pregnancies, which represent 3% of all HL patients [1–7]. In France, the number of new HL cases was estimated to be around 2000 per year, with 45% of them affecting women [8]. Staging the disease helps to determine the appropriate treatment. To treat advanced stages (IIB, III and IV) during pregnancy, the ABVD regimen (doxorubicin, bleomycin, vinblastine and dacarbazine) is considered to be the best therapeutic choice, providing good overall survival and low toxicity for the fetus [9–12]. No consensus has been proposed to treat stage IA and IIA HL during pregnancy, but recommendation is to delay chemotherapy until delivery [11, 12]. Therapeutic decision also depends on gestational age and fetal development. The management of ABVD toxicity requires consideration of physiological changes in pregnancy which affects drug pharmacokinetics. However, based on the limited clinical data about adaptation of chemotherapy during pregnancy, the standard ABVD protocol remains the current treatment for HL in pregnancy in North America and Europe [13, 14]. Small case series and retrospective studies in pregnancies treated for HL reported that doxorubicin was safe for the fetal heart when administered during the second and third trimester [9, 12, 15–17]. But they reported that doxorubicin in pregnancy could reduce the thickness of the left ventricular wall without causing heart defects or functional disorders [15].

In this article, we report a case of HL-complicated a twin dizygotic pregnancy in which ABVD administration was associated with reversible cardiac dysfunction in only one of the twins.

## Case presentation

A 41-year-old woman pregnant with twins was admitted to our hematology department at a gestational age of 27 weeks for stage IIA HL. The disease history started with a vena cava syndrome associated with a right supra-clavicular adenopathy up to 3 cm. A thoracic-abdominal-pelvic scan showed a compressive anterior mediastinal mass of 16 cm in diameter, and a lymph node biopsy confirmed the diagnosis of classic HL. The CRAT (French reference center about teratogenic agents) states that if the examination is necessary to ensure the best diagnostic/treatment of the patient, it can be performed with protective measures in a specialized department. Radiation levels were lower than the maximum recommended during pregnancy [18]. A course of corticosteroid (prednisone 80 mg/d and then 40 mg/d) was started at the time of diagnosis. Maternal transthoracic echocardiography and electrocardiogram were normal. Fetuses’ transabdominal echography at gestational ages of 18 weeks and 4 days, and 22 weeks and 3 days revealed normal morphologic examination. The heart was in normal position and there were four equilibrated cardiac cavities for each of twin. The intersection of the big vessels was normal. No abnormal cardiac rhythm was detectable. Cytological, urinary and hemostasis results were within the normal range at patient admission. A multidisciplinary team (hematologists and obstetricians) decided to start a chemotherapy regimen based on the ABVD protocol at a gestational age of 28 weeks and 3 days. The patient received 2 chemotherapy cycles on a 15-day outpatient basis (Table 1). During each outpatient hematology session, doxorubicin 25 mg/m^2^, bleomycin 10 mg/m^2^, vinblastine 6 mg/m^2^, dacarbazine 375 mg/m^2^ (ABVD), and dexamethasone 10 mg were given. The patient received medications such as ondansetron (8 mg twice a day), prednisone (40 mg per day from the diagnosis until 1 week after the first ABVD), enoxaparin (4000 UI twice a day), lansoprazole (30 mg once a day), and valaciclovir (500 mg once a day). Preterm premature rupture of membranes (PPROM) occurred on the same day as the completion of the second cycle of ABVD, corresponding to a gestational age of 32 weeks and 4 days. She was admitted in the maternity unit for surveillance and was given antibiotics by amoxicillin and steroids for fetal lung maturity. No sign of chorioamnionitis was found at that time (no contractions, no inflammatory sign in blood sample, no fetal tachycardia). She spontaneously went into labor at 33 weeks, and a cesarean section was performed given the prematurity and twin pregnancy. The latency period, from the PPROM to the delivery, seems to be shorter in twins compared with singleton pregnancies. Most studies report a median latency of less than 24 h [19].

**Table 1:** ABVD protocol administered to twin pregnant women

Patient: 41 years old		Weight (kg): 85 Size (cm): 173 Body surface area (m2): 1,99 Creatinine (μmol/L): 58 Clearance(mL/min, Cockroft Gault): 151		Unit: Hematology Cycle 1 J1 = November 08, 2017 Cycle duration: 28 days			
Drug	Dose of protocol	Total dose	Vehicle	Medical device	Route	Duration	Timing
Doxorubicin	25 mg/m2	50 mg	Glucose 5% 20 mL	Syringe	Intravenous	5 min	J1 and J15
Bleomycin	10 mg/m2	19.8 mg	Sodium chloride 0.9% 20 mL	Syringe	Intravenous	5 min	J1 and J15
Vinblastine	5 mg/m2	10 mg	Sodium chloride 0.9% 20 mL	Bag	Intravenous	5 min	J1 and J15
Dacarbazine	375 mg/m2	750 mg	Glucose 5% 250 mL	Bag	Infusion	45 min	J1 and J15
Ondansetron	8 mg	8 mg	No dilution		Intravenous	1 min	J1 and J15
Dexamethasone	10 mg	10 mg	No dilution		Intravenous	5 min	J1 and J15

Monitoring: hemogram, C-reactive protein (CRP), creatininemia, liver checkup

The first twin born was a boy weighing 2030 g (50th percentile) and measuring 48 cm. Head circumference was 31.5 cm, Apgar score was 9/10/10 and umbilical cord pH was 7.32. The neonate presented normal respiration (45 breaths/min) and heart rate was 150 beats/min. Blood count was normal with hemoglobin at 18 g/dL and platelets at 328,000/mm^3^. To avoid premature apnea, an intravenous loading and maintenance dose of caffeine (5 mg/kg/day) was given. The neonate breathed spontaneously with no apnea or bradycardia. Four days after delivery, an echocardiographic control examination showed a minor left cardiac dysfunction with preserved ejection fraction (HF*p*EF) at 48%, associated with a high concentration of troponin (Tn1: 103 ng/L; reference: < 26 ng/L). Cardiological advice was given and no therapy was initiated, but medical monitoring was started. When he was one-month-old, echocardiography findings were normal with a borderline LVEF at 50% without pulmonary arterial hypertension. No troponin measurement was further performed. Following exclusive parenteral nutrition for 4 days, oral nutrition was progressively introduced with complete autonomy on day 17. Neurological investigation was normal.

The second twin was a girl weighing 1785 g (25th percentile) and measuring 42 cm at birth. Head circumference was 32 cm; Apgar score was 9/10/10 and umbilical cord pH was 7.32.Blood count was normal with hemoglobin at 19.2 g/dL and platelets at 310,000/mm^3^. At birth, echocardiography results were normal, with a LVEF > 55%. Neurological investigation was normal.

For the mother, ^18^F-FDG PET and TDM scans were performed 20 days later and revealed complete metabolic remission. She was treated with 6 further ABVD cycles for consolidation.

## Discussion and conclusions

We report here a case of a twin dizygotic pregnancy complicated by HL. A preterm rupture of membranes was diagnosed at 32 weeks and 4 days. ABVD administration was associated with reversible cardiac dysfunction in only one of the twins. Maggen and al. reported in a multicenter cohort study that 3% of 123 children born from mothers diagnosed with HL had a major congenital abnormality (syndactyly, atrial septal defect) at birth and 3% with minor abnormalities. They also reported that mothers who received prenatal chemotherapy experienced more obstetric complications (preterm contractions, preterm ruptures of membranes) [20]. In pregnancy, drug pharmacokinetics may be altered by maternal physiological changes. Thus, maternal drug distribution and/or elimination may be affected. Indeed, maternal serum albumin concentrations decrease from the pre-pregnancy value of 45.8 g/L (7.6%), to 37.6 g/L (10%) at 34 weeks and 31.5 g/L (17%) at 40 weeks of gestation [21, 22]. The increase in intravascular and extravascular fluid partly explains the hypoalbuminemia observed during pregnancy, with a significant increase in the total body water [23]. Hypoalbuminemia can increase the free plasma fraction of drugs and alter their distribution in tissues. However, the low serum protein binding of doxorubicin (~ 75%) limits such variations in doxorubicin distribution.

The CYP2D6 and CYP3A4 activities increase up to 35% during the second and third trimesters [24]. The clearance of doxorubicin (CYP3A4 and CYP2D6 substrate) mainly involves liver metabolization and excretion via the bile duct [21]. However, some reports on doxorubicin clearance during pregnancy provide conflicting results about the effect of pregnancy on doxorubicin metabolism and variations in biliary excretion [22, 25–27]. Doxorubicin, a low molecular weight (544 g/mol) amphiphilic drug is also known as a substrate of the carbonyl reductases CBR1 and CBR3, which convert doxorubicin to doxorubicinol, an active and toxic metabolite, and other enzymes that produce some inactive metabolites (e.g. aglycones, glucuronides, and sulfates) [28–30]. Doxorubicinol is more powerful than doxorubicin to compromise both systolic and diastolic cardiac functions [31]. Cardiotoxicity is reduced by decreasing circulating levels of doxorubicinol through the inhibition of CBR1 [32]. Doxorubicinol formation is under control of AKR1C3, CBR1, and CBR3 expressions [33]. To our knowledge, no data exist about impact of changes in their expression or activity during pregnancy. However, doxorubicinol quantity expressed as a ratio of its plasma concentrations, represented as an area-under-the-curve (AUC) (AUCdoxorubicinol/AUCdoxorubicin: 0.45 ± 0.10; range: 0.33–0.61) was not significantly different from that in non-pregnant women. It suggests that pregnancy does not affect metabolic pathway involved in the doxorubicinol formation [22]. Moreover, these studies also suggest that distribution volume and elimination (half-life) of doxorubicin are not statistically different in pregnant than in non-pregnant women [22, 27–29]. Placental capacity to transfer doxorubicin or pegylated liposomal doxorubicin from maternal blood to the fetal circulation was studied ex vivo with the human placental perfusion model [34]. This study showed a rapid decrease in doxorubicin concentration, with 30% doxorubicin remaining in the maternal circulation after 4 h, and 12% doxorubicin in the fetal circulation. On the contrary, doxorubicin was not detected in the fetal circulation at any time point over 4 h of ex vivo placental perfusion with pegylated liposomal doxorubicin. These results suggest that pegylated liposomal doxorubicin did not cross the blood-placental barrier. While these reported data suggested that the maternal doxorubicin pharmacokinetic did not appear to differ during pregnancy, placental function and fetal factors were also possible sources of variability in the fetal exposure to doxorubicin or in its pharmacokinetics.

To further explain the low risk of fetal doxorubicin cardiotoxicity, the critical role of placental drug efflux transporters of the ABC superfamily in effectively controlling and limiting fetal drug/substrate exposure has been suggested [35, 36]. Some studies demonstrated that ABCB1, also known as P-glycoprotein (P-gp), is critical for fetal protection by limiting exposure to substrates from the maternal circulation and removing them from the fetal circulation to the maternal circulation [37–39]. Table 2 summarizes the localization of selected doxorubicin carrier-mediated systems of the ABC superfamily, known to limit, at least at the human placenta, doxorubicin access to the fetal blood compartment. There are also known to affect the pharmacokinetics of doxorubicin through their polymorphisms. Therefore, the function of these drug transporters may be critical for fetal protection. P-gp and ABCG2 (BCRP) are expressed at the placenta and doxorubicin is well known as a dual P-gp and BCRP substrate. Detected in the syncytiotrophoblast microvilli bordering the maternal side of the human placenta, mean P-gp expression decreases from the 14th to the 40th weeks of gestation, suggesting a gradual decrease in fetal efficacy and protection [35, 36]. Although its clinical consequences are still poorly known, lowered P-gp expression/function at the placenta might increase the distribution of the drug substrate to the fetus, possibly intensifying its effects [37]. P-gp function could also be decreased by drug interactions with inhibitors such as amiodarone, cyclosporine or quinidine [44].

**Table 2:** ABC transporter gene polymorphisms of doxorubicin involved in human placenta [40–43]

Transporter	Syncytiotrophoblast localization	Variant	Modulation on disposition of doxorubicin	Modulation on cardiotoxicity of doxorubicin
ABCB1 (P-gp)	Apical	1236C > T	Higher maximum plasma concentration, higher AUC in T carriers	No data
		Haplotype 1236TT 2677TT 3435TT	Higher plasma concentrations in TT carriers	No data
ABCG2	Apical	421C > A	No significant impact on doxorubicin pharmacokinetics	No data
ABCC1 (MRP1)	Apical	2012G > T	No data	Higher acute cardiotoxicity in T carriers
ABCC2	Apical	3563 T > A 4544G > A	No data	Higher acute cardiotoxicity in A carriers
ABCC5	Basolateral	1679 T > A	Higher doxorubicinol exposure in TT carriers	No data

Intravenous ondansetron to prevent nausea and vomiting was prescribed off-label in our case report. Side effects including potential QT prolongation and torsade de pointes have been reported in the literature [45–47]. In the study conducted by Danielsson et al., data were analyzed from the Swedish medical birth register to identify 1349 infants born from women who had taken ondansetron in early pregnancy. There was a statistically increased risk for cardiovascular defects [48]. The authors hypothesized that potential QT interval prolongation by ondansetron could cause embryonic arrhythmias, suggesting that the drug should not be used off-label in early pregnancy. A recent study conducted by Fejzo et al. analyzed fetal outcome in pregnancies exposed to ondansetron in the United States. The results were similar with these findings [49].

Drug-drug interactions involving doxorubicin metabolism and/or transport should always be considered prior to initiation of doxorubicin treatment. The predictive role of P-gp polymorphism in doxorubicin fetal toxicity requires further clinical studies. Such genetic tests were not performed in our case.

In this case report, the maternal age of 41 years was a risk factor that should be considered. Advanced maternal age can contribute to multiple pregnancy complications such as spontaneous abortion, preeclampsia, gestational diabetes, fetal growth restriction, and stillbirth [50, 51]. Some studies have also reported an increased risk of septal defects in women who become pregnant at an advanced age [52, 53]. Conversely, some studies have not found evidence of an association between maternal age and congenital heart disease [54–56].

One specificity of this case report is that twins reacted differently to the exposure. Even though the twins share the same uterus, the presence of two separate placentas brings a particular environmental influence for each twin [57, 58]. The delivery of compounds, toxic or not, depends on the vascular system devoted to the individual twin [57]. In their study, Igbal and al. suggested that different expressions in the twins-genetic variation in the genes that encode for these drug transporters can significantly alter transporter function and may play a significant role in determining fetal sensitivity to maternal drugs [43]. In 2017, Hermel reported a case in which only one of the twins, born to a mother treated with dasatinib for chronic myeloid leukemia, had cardiac defects [59]. Hence, hypothesis could be placental discrepancy in doxorubicin distribution with a higher blood transfusion for the male twin.

The treatment of HL diagnosed during pregnancy is a major challenge for medical staff, who must propose the best treatment options while avoiding fetal toxicity. We report one case of HL treated with an ABVD regimen during a dizygotic twin pregnancy, complicated by premature rupture of membranes and cardiotoxicity in one of the twins.

Even if no molecular mechanism could be hypothesized in our study, this case highlights the role of specific fetal factors that could confer variability in the pharmacokinetics and/or pharmaco/toxico-dynamics of doxorubicin.

## Data Availability

All data analyzed during this study are included in this published article.

## Abbreviations

ABVD: Adriamycin/doxorubicin, bleomycin, vincristine, dacarbazine
ABC: ATP-binding cassette
AUC: Area under the curve
BCRP: Breast cancer resistance protein; ABCG2
HL: Hodgkin lymphoma
LVEF: Left ventricular ejection fraction
P-gp: P-glycoprotein; ABCB1
